# Sindrome dell’ovaio policistico e infezione da COVID-19

**DOI:** 10.1007/s40619-022-01053-0

**Published:** 2022-03-15

**Authors:** Alessandra Gambineri, Paola Dionese

**Affiliations:** Unità Operativa Complessa di Endocrinologia e Prevenzione e Cura del Diabete, Dipartimento di Scienze Mediche e Chirurgiche, Ospedale S. Orsola, Università di Bologna, Bologna, Italia

Commento a:

**Increased COVID-19 infections in women with polycystic ovary syndrome: a population-based study.**

**A. Subramanian, A. Anand, N.J. Adderley, K. Okoth, K.A. Toulis, K. Gokhale, C. Sainsbury, M.W. O’Reilly, W. Arlt, K. Nirantharakumar.**

**Eur J Endocrinol (2021) 184:637–645**

**COVID-19 – The potential beneficial therapeutic effects of spironolactone during SARS-CoV-2 infection.**

**K. Koftis, K. Lechowicz, S. Drożdżal, P. Niedźwiedzda-Rystwej, T.K. Wojdacz, E. Gzywalska, J. Biernawska, M. Wiśniewska, M. Parczewski.**

**Pharmaceuticals (2021) 14:71–84**

La Sindrome dell’ovaio policistico (PCOS) si caratterizza per essere una patologia iperandrogenica spesso associata a insulino-resistenza e stato infiammatorio cronico di basso grado e frequentemente complicata da obesità e diabete tipo 2, noti fattori di rischio di infezione da COVID-19. Per questo motivo, è stato ipotizzato che la PCOS possa essere una patologia ad aumentato rischio di infezione da COVID-19, dato recentemente confermato in uno studio di popolazione retrospettivo [1]. In questo studio, è stato dimostrato che le donne con PCOS presentano un rischio di infezione da COVID-19 del 51% più elevato rispetto alle donne senza PCOS; tale rischio permane elevato (28%) anche dopo l’aggiustamento per l’obesità e per la ridotta tolleranza glucidica [1]. Le principali cause suggerite dagli autori di aumentato rischio di infezione da COVID-19 nella PCOS sono l’iperandrogenismo e lo stato infiammatorio cronico, entrambe condizioni in grado di facilitare l’instaurarsi di infezioni attraverso una disregolazione del sistema immunitario [1]. Inoltre, l’iperandrogenismo aumenta l’espressione della serina proteasi transmembrana 2 (TMPRSS2), enzima chiave nel mediare l’entrata del virus COVID-19 all’interno delle cellule.

È di interesse notare che l’iperandrogenismo viene oggi considerato anche un fattore di rischio per un’evoluzione verso forme clinicamente più severe di infezione da COVID-19, anche se al momento non ci sono studi che dimostrino che le donne con PCOS si ammalano con maggiore frequenza di forme severe di infezione da COVID-19 rispetto alla popolazione generale. Con questi presupposti, un trattamento antiandrogenico potrebbe ridurre il rischio di infezione da COVID-19 e/o di evoluzione verso forme severe nelle pazienti affette da PCOS. Non sono però al momento disponibili studi specifici che possano supportare questo ipotizzabile beneficio. Ci sono, tuttavia, alcuni interessanti studi che dimostrano come lo spironolattone, farmaco ad attività non solo antiandrogenica, ma anche anti-mineralcorticoide e debolmente anti-infiammatoria e anti-fibrotica, sia in grado di prevenire e trattare le complicanze acute polmonari e, addirittura, di prevenire e di trattare la fibrosi polmonare nell’infezione da COVID-19 [2].

Non sono disponibili studi sull’effetto dello spironolattone nell’infezione da COVID-19 specificatamente nelle donne affette da PCOS. È importante sottolineare, tuttavia, che lo spironolattone, per il buon rapporto rischio/beneficio, è un farmaco ampiamente utilizzato nella donna affetta da PCOS che necessita di un trattamento antiandrogenico, dal momento che determina una buona risposta in termini di miglioramento dell’iperandrogenismo clinico, in particolare l’irsutismo, senza esporre il soggetto trattato a significativi effetti collaterali. Questi sono dei presupposti interessanti per iniziare a studiare l’impiego degli anti-androgeni e dello spironolattone, in particolare nella prevenzione ed evoluzione dell’infezione da COVID-19 nella donna con PCOS.
